# What will it cost to prevent violence against women and girls in low- and middle-income countries? Evidence from Ghana, Kenya, Pakistan, Rwanda, South Africa and Zambia

**DOI:** 10.1093/heapol/czaa024

**Published:** 2020-06-18

**Authors:** Sergio Torres-Rueda, Giulia Ferrari, Stacey Orangi, Regis Hitimana, Emmanuelle Daviaud, Theresa Tawiah, Rebecca Kyerewaa Dwommoh Prah, Rozina Karmaliani, Eleonah Kapapa, Edwine Barasa, Rachel Jewkes, Anna Vassall

**Affiliations:** c1 Department of Global Health and Development, London School of Hygiene & Tropical Medicine, 15-17 Tavistock Place, London WC1H 9SH, UK; c2 Health Economics Research Unit, KEMRI-Wellcome Trust Research Programme, P.O Box 43640 – 00100, 197 Lenana Place, Off Lenana Road, Nairobi, Kenya; c3 School of Public Health, University of Rwanda, Remera Campus, P.O. Box 3286, Kigali KG 11 Avenue, Gasabo, Kigali, Rwanda; c4 Health Systems Research Unit, South African Medical Research Council, Francie Van Zyl Drive, Parow Valley, Cape Town 7503, South Africa; c5 Kintampo Health Research Centre, P.O. Box 200, Kintampo North Municipality, Ghana; c6 School of Nursing and Midwifery and Community Health Sciences, Aga Khan University, National Stadium Road, Karachi 74800, Pakistan; c7 National Institute of Public Administration, P.O Box 31990 Plot No. 4810, Dushanbe Road, Lusaka, Zambia; c8 Nuffield Department of Medicine, University of Oxford, Henry Wellcome Building for Molecular Physiology, Old Road Campus, Headington, Oxford OX3 7BN, UK; c9 Gender and Health Research Unit, South African Medical Research Council, 1 Soutpansberg Road, Pretoria, South Africa

**Keywords:** Costs, violence against women and girls, violence prevention

## Abstract

Violence against women and girls (VAWG) is a global problem with profound consequences. Although there is a growing body of evidence on the effectiveness of VAWG prevention interventions, economic data are scarce. We carried out a cross-country study to examine the costs of VAWG prevention interventions in low- and middle-income countries. We collected primary cost data on six different pilot VAWG prevention interventions in six countries: Ghana, Kenya, Pakistan, Rwanda, South Africa and Zambia. The interventions varied in their delivery platforms, target populations, settings and theories of change. We adopted a micro-costing methodology. We calculated total costs and a number of unit costs common across interventions (e.g. cost per beneficiary reached). We used the pilot-level cost data to model the expected total costs and unit costs of five interventions scaled up to the national level. Total costs of the pilots varied between ∼US $208 000 in a small group intervention in South Africa to US $2 788 000 in a couples and community-based intervention in Rwanda. Staff costs were the largest cost input across all interventions; consequently, total costs were sensitive to staff time use and salaries. The cost per beneficiary reached in the pilots ranged from ∼US $4 in a community-based intervention in Ghana to US $1324 for one-to-one counselling in Zambia. When scaled up to the national level, total costs ranged from US $32 million in Ghana to US $168 million in Pakistan. Cost per beneficiary reached at scale decreased for all interventions compared to the pilots, except for school-based interventions due to differences in student density per school between the pilot and the national average. The costs of delivering VAWG prevention vary greatly due to differences in the geographical reach, number of intervention components and the complexity of adapting the intervention to the country. Cost-effectiveness analyses are necessary to determine the value for money of interventions.

**Key Messages**

- Interventions to prevent violence against women and girls (VAWG) can be delivered in a multiplicity of platforms, through different components, across various settings and with different levels of intensity. The costs involved in delivering VAWG prevention services largely reflect these intervention characteristics.
- In the six interventions costed, the cost per beneficiary ranged widely, from US $4 in a community-based intervention in Ghana to US $1324 for one-to-one counselling in Zambia; cost-effectiveness analyses will determine whether more resource-intensive interventions generate better value for money.
- VAWG prevention interventions are staff intensive. Staff-related costs make up the biggest proportion of costs across all interventions, and total costs are very sensitive to staff salaries. Training frontline staff is a large investment; ensuring retention of staff is essential in maintaining costs low in the long term.
- The cost per beneficiary will likely change when interventions are scaled up from pilots to the national level. Cost modelling suggests that unit costs can decrease in community-based and workshop-based interventions. Conversely, unit costs can increase in interventions with fixed platforms, such as schools, when the average ratio of students-per-school is lower at the national level than in the pilot (due to high fixed costs at the delivery site).

## Introduction

Violence against women and girls (VAWG) is a global problem with profound consequences. Between 30% and 65% of women and adolescent girls over the age of 15 years in sub-Saharan Africa, and over 40% of those in South Asia, have experienced intimate partner violence (IPV) (Devries *et al.*, 2013b). A human rights violation, violence affects women and girls across all socio-economic strata and ages, in both peace and conflict settings (Dartnall and Jewkes, 2013), and has serious public health, social and economic implications (McCollister *et al.*, 2010; Devries *et al.*, 2013a; Duvvury *et al.*, 2013; Stockl *et al.*, 2013).

Governments and international donors, such as the United Kingdom’s Department for International Development (DfID) and the World Bank, have invested in VAWG prevention pilots and programmes (Global Gender-based Violence Task Force, 2017; World Bank, 2017) in low- and middle-income countries (LMICs). The body of evidence on VAWG prevention interventions is rapidly growing and a greater understanding on effective prevention mechanisms across settings, target populations and delivery platforms is emerging (Department for International Development, 2014; What Works to Prevent Violence, 2018). However, the United Kingdom’s Independent Commission for Aid Impact and others have highlighted that the reach of existing VAWG-focused programmes is not commensurate with the magnitude of the challenge (Independent Commission for Aid Impact, 2016). Evidence on the costs of VAWG prevention is critical to support the economic analyses required to justify and plan any scale-up, yet, presently there is almost no evidence on the resource requirements of VAWG prevention to inform policymakers considering investments at scale.

Estimating the costs of public health and social interventions in LMICs requires substantial primary data collection given the dearth of routine financial reporting systems. To date, two economic evaluations of VAWG prevention interventions in LMICs have been published. Evidence on costs and cost-effectiveness are available for the Intervention with Microfinance for AIDS & Gender Equity (IMAGE), a combined microfinance and gender training intervention to prevent IPV in rural South Africa (Jan *et al.*, 2011); and SASA!, a community mobilization intervention in urban Uganda (Michaels-Igbokwe *et al.*, 2016). These studies have suggested that the cost per past person-year free from IPV experience is around 2011 US $460 (adjusted to 2016 US $496) in SASA!. In the case of IMAGE, the intervention was potentially cost-effective, particularly at scale.

What Works to Prevent Violence against Women and Girls (What Works) is a global programme building evidence on violence prevention interventions in 13 LMICs (What Works to Prevent Violence, 2018). The programme provides a unique opportunity to estimate and compare the costs of a multiplicity of delivery platforms and target populations, informing those wishing to scale up VAWG prevention further.

This study has two aims: first, to present the total costs and unit costs of six VAWG prevention interventions piloted through different platforms (communities, workshops for individuals and small groups, schools) and evaluated using randomized controlled trials as part of the ‘What Works’ programme (i.e. costing ‘implementation in a research setting’) in six countries: Ghana, Kenya, Pakistan, Rwanda, South Africa and Zambia; and second, to use these primary data to model the costs of scaling up these interventions at a national level, presenting total costs and unit costs for five of the six countries.

## Methods

### Interventions

We collected the costs of six VAWG prevention interventions in six LMICs. These interventions were piloted and assessed using randomized controlled trials (What Works to Prevent Violence, 2018). Further information on the six trial can be found elsewhere (Gibbs *et al.*, 2017; Kane *et al.*, 2017; McFarlane *et al.*, 2017; Stern *et al.*, 2018; Addo-Lartey *et al.*, 2019; Baiocchi *et al.*, 2019). The interventions varied by delivery platforms: (1) social norms change interventions delivered to communities (Rural Response System, or RRS, in Ghana), (2) workshop-based small-group sessions (Stepping Stones and Creating Futures, or SSCF, in South Africa), (3) classroom-based school interventions (IMPower and Sources of Strength, or IMPower/SOS, in Kenya and the Positive Child and Youth Development Programme delivered by the organization Right to Play, or RTP, in Pakistan), (4) one-to-one sessions on mental health issues (VATU in Zambia) and (5) a combination of workshop-based small-group sessions with couples and a social norms change intervention delivered to communities (Indashyikirwa in Rwanda).

The interventions also varied in terms of setting (urban/rural), coverage (from one city to several regions), theories of change approaches (harmful gender norms change, self-defence, skills and livelihoods building) and durations of implementation (12–31 months). Full information on the interventions costed is available elsewhere (Jewkes *et al.*, 2019), and a summary is presented in Table 1. 

### Costing

To ensure comparability across intervention types, we developed a standardized methodology and a set of guidelines for the economic evaluation of complex (i.e. multi-component and/or multi-platform) programmes designed to prevent VAWG in LMICs (Ferrari *et al.*, 2018; 2019). Our methodology follows best practice established in the Global Health Costing Consortium Reference Case (Vassall *et al.*, 2020) and is consistent with the Second US Panel on Cost-effectiveness in Health and Medicine (Sanders *et al.*, 2016) and the CHEERS guidelines (Husereau *et al.*, 2013), as well as with DfID’s ‘value for money’ framework for the assessment of economy, efficiency, effectiveness and equity of its programmes (Department for International Development, 2011).

We estimated costs of the start-up and implementation phases. The start-up phase was divided into three sub-phases: (1) intervention development, defined as the period when the curriculum or manual of activities was initially designed, (2) adaptation, defined as the process of making the curriculum or manual specific to the target population and (3) set-up, defined as the period of community entry and training of frontline staff. The implementation phase spanned the time between when the first and last clients received the intervention.

All three sub-phases were costed for IMPower/SOS (Kenya), SSCF (South Africa) and VATU (Zambia). For RTP (Pakistan) and Indashyikirwa (Rwanda), only set-up and adaption costs were included. RTP’s development sub-phase started in 2008, and resource-use data were not available; it therefore could not be costed. Indashyikirwa is a multiple-component intervention, partially based on SASA! and Journeys of Transformation (previously developed interventions) but with some newly developed components. Given the complexity of Indashyikirwa’s start-up phase, it was difficult to disaggregate resources used strictly to develop new components vs adaptation of existing components. For analytical simplicity, we classified all costs as adaptation (vis-à-vis development) and tested this assumption in sensitivity analysis. For RRS (Ghana), only the set-up sub-phase was costed. The intervention was developed over 15 years prior to the pilot, and no financial records were available. Furthermore, RRS did not go through an adaptation phase before the implementation.

A full financial and economic micro-costing was carried out for each intervention. Data on resource utilization were obtained through structured interviews, review of financial records, monitoring and evaluations data and travel logbooks. For financial costs, purchase prices were used, with replacement prices for capital goods, commonly available from project records. Costs were separated by phase and sub-phase. Most costs were calculated through a bottom-up approach (measuring quantities in a granular manner at the activity and sub-activity level). When unfeasible, a top-down approach (dividing overall costs by number of outputs) was used to calculate some administration and management costs.

Costs were broken down by input type (e.g. supplies, staff salaries or utilities) and allocated to a number of activities common across interventions (management, administration, technical support, travel, maintenance/cleaning and other) and intervention-specific core activities (e.g. community sensitization or counselling). Research costs were excluded. Implementing organization monitoring and evaluation costs were included as these would be expected to be incurred in routine service delivery. Allocations between activities and sub-activities were based on staff time use and financial and programmatic records (Ferrari *et al.*, 2018; 2019) (see Supplementary Appendix S1 for more information on the sources of resource use and price data for each type of input).

Staff time use was collected through structured interviews, timesheet review and direct observation. When volunteers did not receive a stipend, their time was valued by applying a replacement value determined as the salary of the lowest-tier health worker in each setting (Kasteng *et al.*, 2016). Cost data were disaggregated by delivery site where possible.

Costs were collected in the currency and year in which they were incurred. They were converted to 2016 values using the World Bank gross domestic product (GDP) deflator and to US Dollars using average annual exchange rates (OANDA, 2018). Capital costs were annuitized over the expected life of each item, and a standard 3% discount rate was applied. Start-up costs were treated as a capital item. The useful life of the different sub-phases of the start-up period was estimated to be 10 years for development, 10 years for adaptation and 5 years for set-up. The durations for the development and adaptation sub-phases were estimated by eliciting the expert opinion of senior staff members of the implementing organizations and triangulated against the evidence base in the literature (Jan *et al.*, 2011; Michaels-Igbokwe *et al.*, 2016). For the set-up sub-phase, we assumed the average length of the political cycle, as set-up activities often require political buy-in from leaders in the community and, therefore, may need to be repeated at the start of each political cycle. We tested these assumptions in sensitivity analysis.

### Descriptive cost data analysis

We calculated a total cost, as well as cost per phase, and present results by input type. We calculated three common unit costs across interventions: cost per frontline worker trained, cost per session delivered and cost per beneficiary reached. We acknowledge that the term ‘unit cost’ has various definitions depending on the purpose of the costing and the methodological approach. We defined a unit cost as a mean cost per unit of output, consistent with the Global Health Costing Consortium Reference Case and VAWG-specific guidelines (Ferrari *et al.*, 2019; Vassall *et al.*, 2020). Unit costs were calculated by obtaining the total cost of the core activity (e.g. total costs of training frontline staff), including both direct and indirect costs, and dividing by the total number of units (e.g. total number of volunteers trained). Other unit costs are available from the authors upon request.

The number of beneficiaries was determined with an intention to treat approach, in line with methodological best practice for evaluations alongside trials. For workshop-based interventions (SSCF, VATU), this is the number of beneficiaries enrolled at baseline, irrespective of the number of sessions completed. For community- and school-based interventions (RTP, IMPower/SOS, RRS and Indashyikirwa), which were based on the model of social diffusion where community members (either villagers or pupils in school) were exposed directly or indirectly, the number of beneficiaries was defined as the total number of people within the relevant target population in each cluster (i.e. village or school). Indashyikirwa (Rwanda) had an initial workshop-based component with couples that fed into the community-based approach; however, in this paper, we focus on the beneficiaries at the community level.

### Uncertainty analysis

To account for uncertainty, we carried out a number of one-way deterministic sensitivity analyses. We examined the sensitivity of costs to the following parameters: staff salaries, replacement value of volunteer labour, working days per year, prices of fuel, useful life of adaptation and set-up costs and discount rates (see Supplementary Appendix S2 for details).

There were discrepancies in perceptions of time use between those reported by staff and those reported by the research team in VATU (Zambia) over which full agreement could not be reached. Due to this extra uncertainty, we carried out an additional one-way deterministic sensitivity analysis varying the four parameters around which there was disagreement: percentage of time spent on research activities, percentage of time spent on management activities, amount of time devoted by supervisors to travel, and the inclusion of a one-day feedback session in the start-up phase (see Supplementary Appendix S3). Given the difficulties in separating out development and adaptation costs, we also carried out a one-way deterministic sensitivity analysis for Indashyikirwa (Rwanda) to test the effect on total costs of different assumptions on the distribution between development costs vs adaptation costs (see Supplementary Appendix S4).

### Scale-up analysis

We modelled the costs at national scale in five out of the six interventions. We excluded VATU (Zambia) given the uncertainty about ‘implementation in a research setting’ costs and non-availability of information to model scale-up assumptions.

Cost data from the pilots were used to estimate costs of full-scale intervention delivery at the national level (so-called ‘scale-up’ costs). Scale-up can be conceptualized across different dimensions. The World Health Organization suggests that scaling up can be applied to inputs, outputs, outcomes or impact (World Health Organization, 2008). While the ultimate goal of an intervention is to increase impact, this analysis focuses on the scale-up of inputs required, which at scale may or may not sustain the effectiveness achieved in the pilots.

An increase in inputs at scale requires increased resources (i.e. increased total costs), but costs are not typically a linear function of the number of beneficiaries reached at scale. As interventions are scaled up, some costs remain fixed (i.e. costs that remain constant regardless of the number of outputs) such as the costs of adapting an intervention to a new country, while others should be treated as variable (i.e. costs that vary according to the level of output). Some costs vary as a function of the number of beneficiaries reached (e.g. costs of printing education materials for each beneficiary), while others vary as a function of intermediate outputs, such the number of delivery sites (e.g. costs of training teachers on the intervention in a school).

Moreover, intervention modifications may be necessary when interventions are scaled up from pilot to national levels. Consequently, we consulted with senior members of each implementation team to elicit their expert opinion on the potential scale-up. Specifically, we elicited input on: (1) potential modifications in inputs at scale (e.g. implementer organization staff delivering components of the intervention during pilot vs local teachers at scale), (2) potential intervention delivery modifications at scale (e.g. changes in field worker supervision) and (3) potential magnitude of scale-up according to delivery platform (e.g. 20 middle schools in an urban area in the pilot vs all middle schools in the country). A final list of included modifications, and associated costs, was consequently reviewed and approved by implementers.

A large number of proposed modifications involved changing the staff used to deliver the intervention: in IMPower/SOS (Kenya) service delivery shifted from implementing organization staff to local school teachers; and in Indashyikirwa (Rwanda) training shifted from being led by the implementer organization staff to community volunteers. There was also a reduction in stipends for frontline staff workers (South Africa, Rwanda and Pakistan), a decrease in supervisory activities (Ghana and South Africa), a reduction in the intensity of frontline worker training (Pakistan) and a shortening of the curriculum (Rwanda) between the pilot phase and the national scale-up. We modelled two scenarios: the first with all suggestions incorporated and a second, more cautious, scale-up scenario, where we calculated costs at national scale only accounting for potential changes in inputs. A complete list of assumptions is found in Supplementary Appendix S5.

It should be noted that the impact of modelled changes in inputs or intervention delivery on the interventions’ effectiveness is uncertain; our scale-up costs should therefore be considered exploratory and be monitored carefully during implementation.

We excluded intervention development costs at scale, and adaptation costs were retained but treated as a fixed cost. Set-up costs, which included costs associated with community entry, stakeholder engagement and training of local frontline staff, would need to be incurred for every new delivery site so were assumed to vary as a function of the number of delivery sites.

Implementation costs were divided between those incurred in programme offices (headquarters or regional offices) and delivery sites. Costs of programme offices were divided into direct and indirect costs. Direct costs (related to core programme delivery, technical assistance and transport) were multiplied by the number of delivery sites at scale and indirect costs (related to management, administration, maintenance and cleaning and other) were multiplied by the number of projected programme offices at scale. Average implementation costs per delivery site were multiplied by the estimated number of delivery sites at scale.

Total costs at scale=adaptation costs+(site set up costs×number of delivery sites)+(programme office indirectcosts×number of programme offices)+(programme office direct costs×number of delivery sites)+(site implementation costs×number of delivery sites)

To determine the total number of delivery sites at scale for most interventions, we divided the total number of beneficiaries at scale by the average number of beneficiaries per site in the pilot phase (in other words, we assumed a fixed capacity per site). For IMPower/SOS and RTP, we assumed that the number of sites was determined by the total number of public primary schools in Kenya and by the total number of public middle schools in Pakistan.

The level of expansion of the programme offices necessary at scale was determined by treating indirect costs as step costs (i.e. fixed cost until a threshold is crossed), and we assumed that costs would remain fixed up to the point where the number of delivery sites tripled. After that point, a duplication in the resources needed for indirect activities was considered necessary. This assumption was tested in sensitivity analysis.

To calculate the number of beneficiaries at scale, we first defined the criteria of inclusion in the target population for each pilot interventions (e.g. age range or employment status). We then calculated the total number of people meeting said criteria at the national level. In the cases of community-based interventions in Ghana and Rwanda, this meant all adults age 20–59 years in the country: 12 210 626 and 4 976  600 people, respectively (Ghana Statistical Service, 2012; National Institute of Statistics of Rwanda, 2012). Although these two interventions targeted adults aged 18–60 years, our age criteria of beneficiaries at scale are different due to data availability. For IMPower/SOS, we calculated the total number of children in primary schools in standard grades 5–8 across Kenya, estimated to be 3 311 555 students in 23 584 public primary schools (Kenya National Bureau of Statistics, 2018). For RTP (Pakistan), we calculated the total number of children in middle schools to be 4 057 000 in 16 928 schools (Ministry of Federal Education and Professional Training, Government of Pakistan, 2018). In the case of SSCF, we estimated all unemployed men and women aged 18–30 years in informal settlements across South Africa to be 490 350 people (Housing Development Agency, 2012; Statistics South Africa, 2016, 2018).

## Results

### Pilot programmes

#### Start-up costs

Table 2 shows the total economic start-up costs by sub-phase (across the entire length of the start-up phase). Total costs of the development sub-phase varied between 2016 US $36 742 in SSCF (South Africa) and 2016 US $107 272 in IMPower/SOS (Kenya). Total costs of the adaptation sub-phase ranged from 2016 US $1773 in SSCF (South Africa) to 2016 US $782 973 in Indashyikirwa (Rwanda). Costs of the set-up sub-phase also varied widely, between 2016 US $42 643 in SSCF (South Africa) and 2016 US $1 363 194 in Indashyikirwa (Rwanda).

**Table 2:** Total start-up costs by intervention (2016 US$)

		COMBAT/RRS (Ghana)	IMPower (Kenya)	RTP (Pakistan)	Indashyikirwa (Rwanda)	SSCF (South Africa)	VATU (Zambia)
Intervention development	Total costs		$107 272.13			$36 741.05	$37 815.17
	Years of useful life		10			10	10
	Annuatized cost		$12 575.57			$4307.17	$4433.09
Intervention adaptation	Total costs		$360 113.15	$39 085.10	$782 972.98	$1772.61	$3315.80
	Years of useful life		10	10	10	10	10
	Annuatized cost		$42 216.25	$9163.93	$168 278.58	$207.80	$388.71
Intervention set-up	Total costs	$257 104.14	$109 015.83	$62 685.50	$1 363 194.12	$42 642.71	$191 470.04
	Years of useful life	5	5	5	5	5	5
	Annuatized cost	$56 139.86	$23 804.10	$27 375.33	$545 709.39	$9311.23	$41 808.36

#### Implementation costs

Table 3 shows the total economic costs of implementation (across the entire length of the implementation phase) and their breakdown by input type. Total costs varied greatly: 2016 US $207 523 in SSCF (South Africa), 2016 US $263 138 in IMPower/SOS (Kenya), 2016 US $279 480 in RRS (Ghana), 2016 US $291 091 in RTP (Pakistan), 2016 US $411 665 in VATU (Zambia) and 2016 US $2 788 019 in Indashyikirwa (Rwanda).

**Table 3:** Total implementation costs by intervention (2016 US$)

		COMBAT/RRS (Ghana)		IMpower (Kenya)		RTP (Pakistan)		Indashyikirwa (Rwanda)		SSCF (South Africa)		VATU (Zambia)	
		Cost	% of total costs	Cost	% of total costs	Cost	% of total costs	Cost	% of total costs	Cost	% of total costs	Cost	% of total costs
Capital costs	Equipment	$3334.87	1.19	$1470.64	0.56	$2265.44	0.78	$14 289.34	0.51	$918.14	0.44	$1598.70	0.39
	Buildings: spaces	$17 861.67	6.39	$16 871.32	6.41	$18 713.42	6.43	$63 600.71	2.28	$11 810.85	5.69	$1559.70	0.38
	Buildings: furniture	$1859.03	0.67	$690.08	0.26		0.00	$6360.07	0.23	$741.47	0.36	$1066.19	0.26
	Vehicles	$2025.00	0.72		0.00		0.00	$49 868.04	1.79		0.00	$1238.23	0.30
	Adaptation and set-up	$56 139.86	20.09	$66 020.35	25.09	$36 539.26	12.55	$713 987.97	25.61	$9519.04	4.59	$42 197.07	10.25
Recurrent Costs	Salaried staff: local	$65 200.51	23.33	$130 007.05	49.41	$99 461.12	34.17	$1 043 724.01	37.44	$129 511.86	62.41	$89 582.64	21.76
	Salaried staff: international		0.00		0.00		0.00		0.00		0.00	$197 143.46	47.89
	Volunteer staff	$19 983.68	7.15		0.00	$37 646.62	12.93	$379 363.71	13.61		0.00	$47 073.02	11.43
	Supplies	$22 319.14	7.99	$6720.36	2.55	$36 047.32	12.38	$111 432.68	4.00	$36 550.57	17.61	$16 183.48	3.93
	Building utilities and maintenance	$4328.98	1.55	$3749.37	1.42	$7469.88	2.57	$15 893.15	0.57	$743.04	0.36	$1120.48	0.27
	Transport: vehicle operations (fuel)	$2380.85	0.85	$279.11	0.11	$1127.79	0.39	$17 801.61	0.64		0.00	$2109.62	0.51
	Transport: vehicle maintenance	$690.15	0.25		0.00		0.00	$26 093.29	0.94		0.00	$1273.59	0.31
	Transport: public transportation/rental	$13 912.95	4.98	$23 210.50	8.82	$14 372.10	4.94	$200 409.98	7.19	$17 728.43	8.54	$9518.61	2.31
	Per diems and allowances	$69 443.33	24.85	$14 119.56	5.37	$37 448.09	12.86	$145 194.73	5.21		0.00		0.00
Total		$279 480.03	100.00	$263 138.33	100.00	$291 091.04	100.00	$2 788 019.29	100.00	$207 523.40	100.00	$411 664.80	100.00

The capital costs of implementation (i.e. buildings, vehicles and equipment) were low across all interventions (1–10% of total intervention costs), while recurrent costs made up the majority of total costs (68–89%). Staff costs (including salaried local and international staff and volunteer staff, both those receiving small stipends and those without financial compensation) were the largest cost driver for all interventions, ranging from nearly 30% of total costs in RRS (Ghana) to 81% in VATU (Zambia). In community-based interventions (Ghana and Rwanda), volunteer costs made up 23–27% of staff costs.

The importance of other recurrent costs varied by intervention. Recurrent transport costs (i.e. fuel, vehicle maintenance, public transport/rental) made up between 3% and 9% of total costs across interventions. Per diem and allowances (for both paid staff and volunteers) made up nearly one-quarter of total costs in RRS (Ghana).

#### Unit costs

Table 4 shows unit costs across interventions. The cost per frontline worker trained varied between 2016 US $58 in Indashyikirwa (Rwanda) and 2016 US $272 in RRS (Ghana). The cost per session delivered ranged from 2016 US $5 in RTP (Pakistan) to 2016 US $93 in SSCF (South Africa).

**Table 4:** Unit costs of pilot programmes (2016 US$)

	COMBAT/RRS (Ghana)	IMPower (Kenya)	RTP (Pakistan)	Indashyikirwa (Rwanda)	SSCF (South Africa)	VATU (Zambia)
Cost per front line worker trained	$272	$220	$70	$58	$211	$191
	*N* = 122	*N* = 99	*N* = 21	*N* = 840^a^	*N* = 10	*N* = 45
Cost per session delivered	$60	$77	$5	$17	$93	b
	*N* = 584	*N* = 3328	*N* = 11 443	*N* = 20 160^a^	*N* = 801	
Cost per participant reached	$3.79	$10.94	$18.23	$17.38	$306.53	$1323.68
	*N* = 73 759	*N* = 24 055	*N* = 15 968	*N* = 141 733^a^	*N* = 677	*N* = 311

a Refers to community activism component.

b Final numbers of total number of sessions delivered not provided by implementing organization.

There was a wide range of costs per participant reached: 2016 US $4 in RRS, a community-based intervention in Ghana, 2016 US $11 in IMPower/SOS, a school-based intervention in Kenya, 2016 US $17 in Indashyikirwa, a couples and community-based intervention in Rwanda, 2016 US $18 in RTP, a school-based intervention in Pakistan, 2016 US $307 in SSCF, a small group intervention in South Africa and 2016 US $1324 in VATU, a largely one-to-one intervention in Zambia.

#### Uncertainty analysis

**Figure 1:**
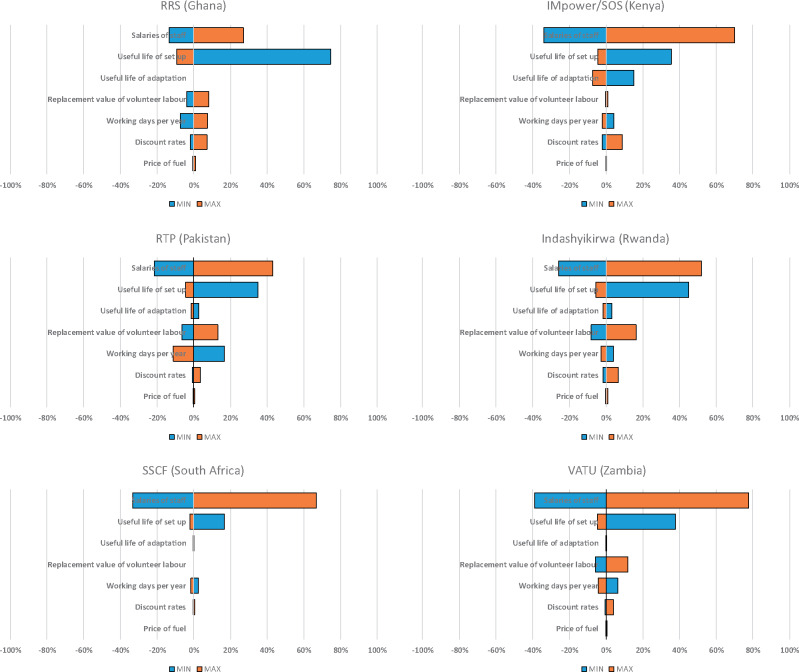
Tornado diagrams of percentage changes to total costs from deterministic one-way sensitivity analysis of key input variables per country.

Deterministic sensitivity analyses are presented in Figure 1. Total costs were most sensitive to changes in staff salaries in all interventions except RRS (Ghana). Staff salaries were particularly important in VATU (Zambia) where a doubling in salaries would increase total costs by over 77%; reducing them by half would reduce total costs by over 38%. The useful life of set-up activities (e.g. community entry and training) was also an important driver, particularly in RRS (Ghana), Indashyikirwa (Rwanda) and RTP (Pakistan), where assuming a useful life of set-up of 1 year (as opposed to a base case of 5 years) would increase total costs by 75%, 45% and 35%, respectively. For VATU (Zambia), the additional uncertainty generated by differences in staff and research programme reported time use resulted in potential changes in total costs of between −13.5% and 13.5%. Likewise, for Indashyikirwa (Rwanda), we found that, if all relevant costs were assumed to be related to development instead of adaptation, there would be a reduction in overall costs of 4% (see Supplementary Appendices S3 and S4).

### Scale-up costs

Table 5 shows the total economic costs and cost per beneficiary reached in two scenarios of national scale-up: the first takes into account potential changes in inputs and modifications to the intervention and the second only takes into account changes in inputs.

**Table 5:** Total and unit cost estimates at scale-up (2016 US$)

		COMBAT/RRS (Ghana)	IMPower (Kenya)	RTP (Pakistan)	Indashyikirwa (Rwanda)	SSCF (South Africa)
Pilot scale: changes in inputs (Total costs )	Start-up costs	$56 139.86	$66 778.50	$36 539.26	$620 936.39	$9519.04
	Implementation costs	$223 340.16	$197 117.98	$217 704.81	$2 074 031.31	$188 650.68
	Total costs	$279 480.03	$263 896.47	$254 244.07	$2 694 967.70	$198 169.72
Pilot scale: intervention modifications (Total	Start-up costs	$56 139.86	$66 778.50	$36 109.01	$533 783.46	$9519.04
	Implementation costs	$199 800.49	$197 117.98	$217 704.81	$2 074 031.31	$174 998.45
costs)	Total costs	$255 940.35	$263 896.47	$253 813.10	$2 607 814.78	$184 517.49

Scenario 1: national scale-up (incl. changes in inputs and intervention modifications) (Total costs)	Start-up costs	$9 293 820.29	$10 769 550.87	$22 815 550.79	$14 321 736.74	$6 744 318.98
	Implementation costs	$23 082 928.11	$75 801 532.93	$145 310 193.75	$40 592 264.69	$97 544 072.20
	Total costs	$32 376 748.40	$86 571 083.79	$168 125 744.53	$54 914 001.43	$104 288 391.17
Scenario 1: number of beneficiaries at national scale-up		12 210 626	3 311 555	4 057 000	4 563 077	490 350
Scenario 1: cost per beneficiary at national scale-up		$2.65	$26.14	$41.44	$12.03	$212.68
Scenario 1: changes in cost per beneficiary between pilot projects and national scale-up (%)		−30.02	+238.43	+227.33	−36.71	−30.62

Scenario 2: national scale-up (incl. changes in inputs only) (Total costs)	Start-up costs	$9 293 820.29	$10 769 550.87	$23 179 644.38	$17 127 608.30	$6 744 318.98
	Implementation costs	$26 914 806.95	$75 801 532.93	$145 310 193.75	$40 592 264.69	$107 282 331.88
	Total costs	$36 208 627.24	$86 571 083.79	$168 489 838.12	$57 719 872.99	$114 026 650.86
Scenario 2: number of beneficiaries at national scale-up		12 210 626	3 311 555	4 057 000	4 563 077	490 350
Scenario 2: cost per beneficiary at national scale-up		$2.97	$26.14	$41.53	$12.65	$232.54
Scenario 2: changes in cost per beneficiary between pilot projects and national scale-up (%)		−21.74	+238.43	+227.82	−33.47	−24.14

Total costs of national scale-up varied widely. For the first scenario, the total costs at scale were: 2016 US $32.4 million for RRS (Ghana), 2016 US $54.9 million for Indashyikirwa (Rwanda), 2016 US $86.6 million for IMPower/SOS (Kenya), 2016 $US 104.3 million for SSCF (South Africa) and 2016 $US 168.1 million for RTP (Pakistan). For the second scenario, the total costs at scale were: 2016 US $36.2 million for RRS (Ghana), 2016 US $57.7 million for Indashyikirwa (Rwanda), 2016 $US 114 million for SSCF (South Africa) and 2016 US $168.5 million for RTP (Pakistan). Total costs were the same in both scenarios for IMPower/SOS (Kenya) as no modifications to service delivery were assumed.

Total costs have been broken down to show cost changes due to suggested modifications to inputs, modifications to service delivery and delivery at scale. Suggested input changes in RTP (Pakistan), SSCF (South Africa) and Indashyikirwa (Rwanda) led to decreases in total costs (13%, 5% and 3%, respectively) and to a minor increase in IMPower/SOS (Kenya) (<1%). No changes in inputs were suggested for the intervention in Ghana.

Changes in service delivery led to further reductions in total costs in the other interventions: 8% in Ghana (reduction in supervision), 7% in South Africa (reduction in meetings with facilitators and senior facilitators), 3% in Rwanda (reduction in training; training carried out by volunteers instead of staff members) and <1% in Pakistan (reduction in training). No changes in delivery were suggested for the intervention in Kenya.

Unit costs per beneficiary reached at national scale in the first scenario were found to be 2016 US $2.65 in RRS (Ghana), 2016 US $12.03 in Indashyikirwa (Rwanda), 2016 US $26.14 in IMPower/SOS (Kenya), 2016 US $41.44 in RTP (Pakistan) and 2016 US $212.68 in SSCF (South Africa). Unit costs in the second scenario were 2016 US $2.97 in RRS (Ghana), 2016 US $12.65 in Indashyikirwa (Rwanda), 2016 US $41.53 in RTP (Pakistan) and 2016 US $232.54 in SSCF (South Africa). Unit costs did not change between the two scenarios in IMPower/SOS (Kenya).

Unit costs at scale were lower than those calculated in the pilot projects across all interventions, except for the two school-based ones. The cost per beneficiary reached decreased by 30%, 31% and 37% for RRS (Ghana), SSCF (South Africa) and Indashyikirwa (Rwanda), respectively, in the first scenario and 22%, 24% and 33%, respectively, in the second scenario. On the contrary, unit costs more than doubled for RTP (Pakistan) and IMPower/SOS (Kenya) in both scenarios.

We assumed that indirect costs at the programme office-level would double for every 3-fold increase in the number of delivery sites. A proportional increase in indirect costs based on the number of sites (i.e. doubling indirect costs for every 2-fold increase in sites) increased total costs at scale by between 0.1% in IMPower/SOS (Kenya) and 0.7% in Indashykirwa (Rwanda). Conversely, a doubling of indirect costs for every 10-fold increase in number of sites decreased total costs at scale from 0.1% in RRS (Ghana) and RTP (Pakistan) to 2.1% in Indashyikirwa (Rwanda).

## Discussion

Our study presents the total costs and unit costs of developing, adapting, setting up and implementing six different interventions to prevent VAWG and the modelled costs of scale-up of five interventions. This is the first comprehensive cost dataset for VAWG prevention, and it demonstrates the application of a method for estimating costs in line with global health costing reference cases. The interventions costed vary in terms of delivery platforms, methods and target populations. Given the dearth of evidence in this area, our work represents a sizeable and valuable contribution for decision-makers and policymakers wishing to plan and advocate the scale-up of VAWG prevention programmes globally.

We divided start-up costs into three sub-phases: intervention development, adaptation and set-up, to inform other pilots and the expansion of services across countries. Development was costed in Kenya, South Africa and Zambia (in all cases at least some of the development occurred prior to ‘What Works’) and costs ranged from ∼2016 US $37 000 to 2016 US $107 000. These figures are higher than those reported for IMAGE (2004 US $13 877 adjusted to 2016 US $17 353) and lower than those reported for SASA! (2011 US $139 000 adjusted to 2016 US $149 944). There was a wider range in adaptation costs: from ∼2016 US $1800 SSCF (South Africa) to 2016 US $783 000 in Indashyikirwa (Rwanda), reflecting different intensities in the process. Whereas, in South Africa, adaptation involved 1 day of post-pilot work in making content and delivery more context specific, the process of adapting SASA! to Rwanda was complex and time-consuming (Stern *et al.*, 2018). As an example, a month-long curriculum pre-testing phase, where trainings were followed by focus groups, led to substantial changes to the programme, including additional training time, modification of conceptual framework to fit local standards and extended provision of psychosocial support for staff.

Whether the high cost of adaptation is warranted is difficult to determine. Ensuring that interventions are context specific is particularly important for interventions targeting social norms. If existing social norms are not understood by implementers, programmes that aim to change them may not be successful. For example, messaging from SASA! was adjusted to the Rwandan social context (e.g. changing emphasis of activism material from HIV to economic empowerment) and to reflect changes in the intervention (e.g. more explicit emphasis on couples taking action together).

There was also a wide range of set-up costs across the interventions, from ∼2016 US $43 000 in SSCF (South Africa) to 2016 US $1 363 000 in Indashyikirwa (Rwanda). Our set-up cost estimates are in all cases (except for SSCF in South Africa), higher than those published in the literature; total cost estimates for set-up for IMAGE were 2004 US $61 000 (adjusted to 2016 US $76 280) in the trial phase and 2004 US $58 000 (adjusted to 2016 US $72 529) in the scale-up, although these costs only include training and exclude community entry costs.

Our set-up costs include training and other community entry activities, such as recruitment of frontline staff, meetings with stakeholders and demand creation, all of which have to be replicated in each geographical area where the implementation occurs. The high costs in Rwanda can be partially explained by Indashyikirwa’s wide geographical reach: unlike the other interventions, which were rolled out in one or two districts or cities, Indashyikirwa was implemented across seven districts in three regions of the country. Indashyikirwa also required the training of four distinct cadres of volunteers per district (couples, community activities, women’s safe space facilitators and opinion leaders); in other interventions, only one type of frontline staff was recruited and trained. Lastly, Indashyikirwa was set up and implemented by three non-profit organizations, each with specific overhead cost structures.

We made an assumption that the useful life of the set-up phase was 5 years, based on an average political cycle. When tested in sensitivity analysis, costs were sensitive to this assumption in Ghana, Rwanda and Pakistan, where community members were trained to deliver services, a process requiring substantial investment. Avoiding attrition of volunteers may be an important factor in ensuring a longer useful life of the set-up sub-phase and, consequently, maintaining lower costs in the long term.

The cost per frontline worker trained varied between 2016 US $58 in Indashyikirwa (Rwanda) and 2016 US $272 in RRS (Ghana), both interventions where volunteers were trained to carry out community outreach activities. However, the unit cost for Indashyikirwa is potentially underestimated; this figure only includes the costs of community activism training and excludes costs of a prior workshop-based training with couples, from which the community activists were subsequently selected (however, the costs of the couples’ training is included in the total set-up costs). Should funders consider implementing only a sub-selection of components, further work could explore costs disaggregated by activity in multi-component interventions.

The cost per beneficiary-facing session delivered also varied between 2016 US $5 per school session in RTP (Pakistan) and 2016 US $93 in SSCF (South Africa). Differences in cost can be explained by the fact that sessions in Pakistan were delivered by volunteers with relatively low stipends and were short in duration, whereas in SSCF the target audience was young adults, requiring more intensive support in livelihoods development.

The cost per beneficiary reached varied greatly: between ∼2016 US $4 in RRS (Ghana) and 2016 US $1324 in VATU (Zambia). Cost per beneficiary in Indashyikirwa were within the same range as in SASA! (2011 US $15–23 adjusted to 2016 US $16–25 in the latter). The wide range in unit costs are primarily explained by the intensity of interaction and staff type required to deliver each intervention and, in particular, by the frontline staff-to-beneficiary ratio. More resources are needed to teach a small number of people (or one person) vs holding public, open-air activities for, at times, hundreds of people at once.

We have presented the modelled total and unit costs of the interventions’ national scale-up. Cost per beneficiary reached ranged from 2016 US $3 in RRS (Ghana) to 2016 US $233 in SSCF (South Africa). The costs per beneficiary reached at scale were lower than those in pilot programmes in three interventions: RRS (Ghana), Indashyikirwa (Rwanda) and SSCF (South Africa). The only current ex-post evidence of scale-up costs comes from IMAGE, which was expanded from the trial setting covering 855 women (in four study villages) to 3453 within the local area; the cost per participant reached fell by ∼70% (from 2004 US $43, adjusted to 2016 US $54 to 2004 US $13, adjusted to 2016 US $16). Comparatively more modest decreases (22–37%) were estimated in RRS, Indashyikirwa and SSCF. However, in IMAGE, expansion occurred within the same geographical area as the pilot and so additional costs required were proportionally smaller. In our model, expansion occurs nationwide.

The reduction in unit costs in the scale-up scenario in three of our interventions is in line with potential economies of scale: unit costs decrease as unit of outputs increase due to the smoothing of fixed costs across a greater number of units (Guinness *et al.*, 2005; Kumaranayake, 2008). However, the cost per beneficiary increased substantially in both school-based interventions: IMPower/SOS (Kenya) and RTP (Pakistan). This increase can be explained by the cost structure of the interventions, delivery platforms and the number of beneficiaries reached. In the other three interventions, we assumed a set number of beneficiaries per site using pilot data and the number of sites at scale was estimated proportionally. In the case of IMPower/SOS and RTP, the number of sites at scale was predetermined by the number of existing schools in the country, regardless of the number of students in each school. Whereas the pilot interventions were implemented in high-density urban areas, with an average of 444 eligible students per school in Kenya and 798 in Pakistan, at a national level, the average number of eligible students per school was 140 and 240, respectively. In addition, in the case of IMPower/SOS, the changes in inputs assumed at scale increased total costs (<1%), as the assumed salary of a teacher was higher than that of the implementer organization staff delivering the intervention at pilot level.

Some have speculated that VAWG prevention interventions could be delivered at scale in an efficient manner given large fixed costs (e.g. developing training curricula) (Remme *et al.*, 2015). However, the degree of efficiency at scale depends on the delivery platform. While other interventions, such as media campaigns, may incur small incremental scale-up costs, labour-intensive interventions require greater increases in variable costs and may not be as efficient at scale.

Given that the majority of costs are fixed at the delivery site level, a greater number of beneficiaries per site is will decrease unit costs at scale. Community-based interventions can have more flexible delivery platforms and therefore programmes could set the number of cadres of front-line staff proportionally to the size of the adult population of a specific geographical area to meet target unit costs. Small group-based interventions could see reductions in unit costs by increasing the number of participants per site, although this needs to be weighed against potential changes in the quality of the programme stemming from different tutor-to-participant ratios.

School-based interventions and other interventions relying on existing institutional infrastructures, on the other hand, will have a fixed number of beneficiaries. To reduce unit costs, programmes could focus on high-density areas, although this would penalize low-density and hard-to-reach communities. Furthermore, the co-delivery of other interventions within the same school platforms (such as health interventions) could help IMPower/SOS and RTP lower unit costs and achieve economies of scope (i.e. reductions in unit costs as the types of services increase). In addition, in schools with fewer students, facilitators could deliver each session to more than one class at a time, thus reducing the cost per student reached.

Alternatively, different models of delivery could shift activities from volunteers to school teachers, as has been done in other settings by the implementers of RTP in Pakistan. However, this model has not been evaluated in a trial setting and, therefore, there is uncertainty around its effectiveness. A shift from head coaches to teachers could reduce financial costs for the implementing organization; whether this reduces or increases economic costs depends on the degree to which teachers have idle time or whether VAWG prevention activities displace other teaching duties creating a need for further staffing.

## Limitations

Our study suffers from several limitations. Development costs were not captured in three of the interventions costed as records were unavailable. While we report total development costs where available, we exclude annuitized development costs from implementation calculations to ensure comparability. It was difficult to differentiate between development and adaptation costs in Indashyikirwa. However, sensitivity analysis showed that assumptions on the allocation of these costs had little effect on total costs.

We collected costs at different time points, and some data were captured retrospectively. While not problematic when reviewing financial records, capturing time use data post-facto through interviews can be subject to recall bias.

In our scale-up analysis, we assume that costs of adaptation will not be replicated when the intervention is scaled up within the same country. However, this presupposes a high degree of in-country homogeneity, which varies by setting.

Furthermore, we model the costs of expanding each intervention in the country in which it was trialled. However, scale-up may prove more complex, involving a combination of different interventions targeting different types of beneficiaries through different platforms depending on need (e.g. implementing community-based interventions only in rural areas and school-based ones only in cities).

Our data could be used to plan scaling up programmes in the countries where they were piloted. However, while they could also be used to estimate costs in other countries, it is important to reflect on issues of transferability. We have already mentioned the high adaptation costs involved in Indashyikirwa; whether further adaptations in other countries would be as costly is uncertain. Staff costs made up the majority of total costs in our study and, thus, understanding how salaries vary between countries is important. Furthermore, these interventions require different types of skilled and semi-skilled workers who may not be readily available in some countries, potentially increasing training costs. Changes to the intervention’s length and intensity of treatment, which would have cost implications, could also potentially be necessary depending on the receptiveness of the target population. Costs at scale may also vary in other countries depending on the availability of similar delivery platforms.

Our work provides a substantive contribution to the economic literature on VAWG prevention but further work is needed. While unit costs are relevant for purposes of budgeting and ensuring financial sustainability, they do not explain whether the intervention has ensured good value for money. More resource-intensive interventions (such as those delivered to smaller groups) may be more effective; and so cost-effectiveness analyses are required to shed light on whether the additional costs represent good value for money.

As part of the process of calculating scale-up costs, we engaged implementers to propose service adaptations potentially implementable at scale. However, whether changes to programme inputs and delivery could lead to changes in effectiveness is unknown. Further empirical work is needed to see if our estimated costs are achieved at scale and understand the trade-off between reduced costs in programme delivery and sustained effectiveness in VAWG prevention interventions. However, if resources required to carry out empirical work, particularly trials, are not available, other techniques such as decision analytic modelling could be considered.

While our analysis gives an indication of changes in unit costs at scale, additional costs needed to deliver services to the hard to reach are not explicitly modelled. Further work is needed to understand the optimal scale to which service delivery should be extended. Moreover, although increasing financial, human and capital resources is a necessary part of scaling up interventions, these increases on their own may not guarantee a successful scale-up (World Health Organization, 2008). Creation of adequate demand, strategic sequencing of expansion and engagement with local stakeholders and institutions may also be necessary and may require additional resourcing.

## Conclusions

VAWG presents a substantial human rights and societal challenge globally. Interventions that prevent VAWG demonstrate a wide range of costs, varying according to the geographical reach, number of intervention components, platforms and the complexity of adapting the intervention to the country. ‘What Works’ has proved a unique opportunity to estimate and compare these costs using standardized methods across different intervention models, providing a substantial source of costs to assist those planning the scale-up of VAWG interventions going forward.

## Supplementary data

Supplementary data are available at *Health Policy and Planning* online.

**Table 1:** Typologies of pilot interventions costed

Intervention name	RRS	IMPower/SOS	RTP	Indashyikirwa	SSCF	VATU
Country	Ghana	Kenya	Pakistan	Rwanda	South Africa	Zambia
Setting	Rural	Urban (informal settlements)	Urban	Rural	Urban (informal settlements)	Urban
Location	Central region (two districts)	Nairobi	Hyderabad (Sindh province)	Eastern, Northern, Western provinces (seven districts)	Durban	Lusaka
Target population	Adults in the community (mostly aged 18–60 years)	Children primary schools (grades 5–8)	Children primary schools (grades 6–8)	Adults in the community (mostly aged 18–60 years)	Unemployed men and women aged 18–30 years in informal settlements	Adult men, with alcohol and other substance abuse issues and VAWG, and their female partners and children
Platform of delivery	Community-based	School based to classes after school	School based to classes during school	Community based and small groups	Small groups	One-on-one sessions
Implementing organization(s)	Gender centre	Ujamaa	RTP Pakistan	CARE Rwanda, Rwanda Women’s Network and RWAMREC	Project empower	SHARPZ, Johns Hopkins University
Approach	Addressing harmful social norms on gender and violence	Self-defence	Play-based life skills	Addressing harmful social norms on gender and violence	Gender transformative and livelihoods strengthening	Psychotherapeutic
Number of sites (intervention arm)	20 communities	52 schools	20 schools	14 sectors	16 sites	3 sites (123 families)
Number of beneficiaries	73 759	24 055 (school children and children in the community)	15 968	141 733	677	311
Start-up phase	January–December 2016	October 2009–March 2016	January 2015–February 2018	October 2015–May 2016	December 2011–December 2015	September 2015–May 2016
Implementation phase	December 2016–December 2017	January–December 2016	November 2015–February 2018	September 2016–July 2018	January 2016–March 2017	June 2016–December 2017

## Supplementary Material

czaa024_supplementary_data
