# Supplementary material for: What will it cost to prevent violence against women and girls in low- and middle-income countries? Evidence from Ghana, Kenya, Pakistan, Rwanda, South Africa and Zambia
Source: Health Policy Plan. 2020 Jun 18;35(7):855–66. doi: 10.1093/heapol/czaa024 (PMC7487331; doi:10.1093/heapol/czaa024)
Supplement: czaa024_supplementary_data [file czaa024_supplementary_data.docx]

**Appendix 1: Sources of Resource Use and Price Data**

|  | **Resource Use** | **Price** |
| --- | --- | --- |
| **Equipment** | Based on staff time allocation; if equipment had single user (e.g. laptop), the individual's time allocation was used; if equipment was of communal use (e.g. office printer) an average staff allocation was used | Implementing organisation's asset register |
| **Building: spaces** | Based on average staff allocation | Implementing organisation financial records |
| **Building: furniture** | Based on average staff allocation | Implementing organisation financial records |
| **Vehicles** | Allocated to intervention as per resource use of vehicle operations (fuel) | Implementing organisation's asset register |
| **Salaried staff: local** | Structured interviews, timesheet review and direct observation | Individual salary information from implementing organisation |
| **Salaried staff: international** | Structured interviews, timesheet review and direct observation | Individual salary information from implementing organisation; when unavailable comparable international prices |
| **Volunteer staff** | Structured interviews, timesheet review and direct observation | Stipends; if no stipends, shadow prices (lowest-level health worker) |
| **Supplies** | Structured interviews; Implementing organization financial records | Implementing organisation financial records |
| **Building utilities and maintenance** | Based on average staff allocation | Implementing organisation financial records |
| **Transport: vehicle operations (fuel)** | Vehicle-specific log books detailing mileage and purpose of individual trips | Implementing organisation financial records |
| **Transport: vehicle maintenance** | Allocated to intervention as per resource use of vehicle operations (fuel) | Implementing organisation financial records |
| **Transport: public transportation/rental** | Implementing organization financial records | Implementing organisation financial records |
| **Per diems/allowances** | Implementing organization financial records; log books; attendance sign-in sheets | Implementing organisation financial records |

**Appendix 1: Parameters of Deterministic Sensitivity Analysis**

|  | | **Base Value** | **Minimum Value** | **Maximum Value** |
| --- | --- | --- | --- | --- |
| **COMBAT/RRS  (Ghana)** | **Salaries of staff** | Variable | x0.5 | x2 |
|  | **Replacement value of volunteer labour and stipends** | 700 GHS/month | 350 GHS/month | 1400 GHS/month |
|  | **Working days per year** | 264 | 211 | 317 |
|  | **Price of fuel (average)** | 4.5 GHS/litre | 2.25 GHS/litre | 9 GHS/litre |
|  | **Useful life of adaptation** | 10 | 5 | 20 |
|  | **Useful life of set up** | 5 | 1 | 10 |
|  | **Discount rates** | 3% | 1% | 10% |
| **IMpower  (Kenya)** | **Salaries of staff** | Variable | x0.5 | x2 |
|  | **Replacement value of volunteer labour and stipends** | -- | -- | -- |
|  | **Working days per year** | 250 | 201 | 301 |
|  | **Price of fuel (average)** | Variable | x0.5 | x2 |
|  | **Useful life of adaptation** | 10 | 5 | 20 |
|  | **Useful life of set up** | 5 | 1 | 10 |
|  | **Discount rates** | 3% | 1% | 10% |
| **RTP (Pakistan)** | **Salaries of staff** | Variable | x0.5 | x2 |
|  | **Replacement value of volunteer labour and stipends** | Variable | x0.5 | x2 |
|  | **Working days per year** | 252% | 202 | 302 |
|  | **Price of fuel (average)** | 74 PKR/litre | 37 PKR/litre | 148 PKR/litre |
|  | **Useful life of adaptation** | 10 | 5 | 20 |
|  | **Useful life of set up** | 5 | 1 | 10 |
|  | **Discount rates** | 3% | 1% | 10% |
| **Indashyikirwa  (Rwanda)** | **Salaries of staff** | Variable | x0.5 | x2 |
|  | **Replacement value of volunteer labour and stipends** | Variable | x0.5 | x2 |
|  | **Working days per year** | 264 | 211 | 317 |
|  | **Price of fuel (average)** | 1100 RWF/litre | 550 RWF/litre | 2200 RWF/litre |
|  | **Useful life of adaptation** | 10 | 5 | 20 |
|  | **Useful life of set up** | 5 | 1 | 10 |
|  | **Discount rates** | 3% | 1% | 10% |
| **SSCF  (South Africa)** | **Salaries of staff** | Variable | x0.5 | x2 |
|  | **Replacement value of volunteer labour and stipends** | -- | -- | -- |
|  | **Working days per year** | 250 | 200 | 300 |
|  | **Price of fuel (average)** | -- | -- | -- |
|  | **Useful life of adaptation** | 10 | 5 | 20 |
|  | **Useful life of set up** | 5 | 1 | 10 |
|  | **Discount rates** | 3% | 1% | 10% |
| **VATU  (Zambia)** | **Salaries of staff** | Variable | x0.5 | x2 |
|  | **Replacement value of volunteer labour and stipends** | Variable | x0.5 | x2 |
|  | **Working days per year** | 250 | 200 | 299 |
|  | **Price of fuel (average)** | 12 ZMW/litre | 6 ZMW/litre | 23 ZMW/litre |
|  | **Useful life of adaptation** | 10 | 5 | 20 |
|  | **Useful life of set up** | 5 | 1 | 10 |
|  | **Discount rates** | 3% | 1% | 10% |

**Appendix 2: Assumptions on National Scale Up**

| Intervention | Changes in inputs | Intervention modifications | Magnitude of scale up/delivery platform |
| --- | --- | --- | --- |
| RRS  (Ghana) | No changes | **Number of supervisory visits**  **Trial:** 4 yearly supervisions from implementing organisation  **Scale up:** 2 yearly supervisions | Expansion through local community leaders in all districts of the country. A COMBAT team was assumed to cover a population of 3689 eligible adults. |
| IMpower/SOS  (Kenya) | **Instructors:**  **Trial:** Implementer organization instructors delivered trainings **Scale up:** Train teachers to deliver instead | No changes | Expansion through the same platform as trial (schools). Assuming expansion to over 23,584 primary schools (public) across the country. |
| RTP  (Pakistan) | **School Coaches**  **Trial:** A head coach covers one school, which has one set of toys and equipment **Scale up:** A head coach covers two schools and shares play equipment between the two | **Time of coach training:**  **Scale up:** A reduction of 20% | Expansion through the same platform. Assuming expansion to all 16,928 public middle schools across the country. |
| Indashyikirwa  (Rwanda) | **Couples and activists’ stipends:**  **Trial:** Received stipend **Scale up:** Stipend removed if services co-delivered through a conditional cash transfer programme | **Trainers:**  **Trial:** Training of couples and activists done by field officers of NGOs **Scale up:** Carried out by volunteers from National Women Councils  **Number of sessions for couples:**  **Trial:** 22 sessions **Scale up:** 18 sessions | To expand, the intervention could be co-delivered through a government programme, such as the conditional cash transfer programme, or Izu, Village Agents Network, Parents Evening Network, or other programmes that are present across every district. A team of community activists, women space facilitators and opinion leaders was assumed to cover a total population of 9449 eligible adults. |
| SSCF  (South Africa) | **Salary of facilitators:**  **Scale up:** A reduction in salary by 38%  **Salary of senior facilitators:**  **Scale up:** A reduction in salary by 19%  **Cost of meal allowances:**  **Scale up:** A reduction by 22%  **Cost of transport allowances:**  **Scale up:** A reduction by 25% | **Meetings with facilitators:**  **Trial:** Weekly one-day meetings throughout **Scale up:** Weekly one-day meetings for 4-6 weeks, then switch to monthly one-day meetings.  **Senior facilitator supervision:**  **Trial:** They visit groups approximately once per week **Scale up:** Monthly visits | Target population continues to be unemployed men and women (18-30) in informal settlements in urban areas but programme is expanded from one city to all urban areas in the country. Facilitators were assumed to cover 52 eligible adults per site. |

**Online Appendix 1: Additional Deterministic Sensitivity Analysis for VATU (Zambia)**

**Table 1: Parameters for Sensitivity Analysis**

|  | | **Base Value** | **Minimum Value** | **Maximum Value** |
| --- | --- | --- | --- | --- |
| **VATU (Zambia) (Ghana)** | **Percentage of total costs spent on research (excluded from total costs)** | Start up phase: 31.88% Implementation phase: 39.45 % | -25% Start up phase: 23.91% Implementation phase: 29.59% | +25% Start up phase: 39.86% Implementation phase: 49.31% |
|  | **Percentage of total costs spent on administrative tasks** | Start up phase: 9.80% Implementation phase: HQ 20.86%; Sites 9.26% | -25% Start up phase: 7.35% Implementation phase: HQ 15.51%; Sites 6.95% | +25% Start up phase: 12.25% Implementation phase: HQ: 25.85%; Sites 11.58% |
|  | **Average percentage of supervisor time spent traveling** | Start up phase: 8.34% Implementation phase: 8.65% | -25% Start up phase: 6.26% Implementation phase: 6.49% | +25% Start up phase: 10.43 % Implementation phase: 10.81% |
|  | **Inclusion of one-day feedback as part of the adaptation sub-phase (2016 US$)** | $ 3,315.80 | 0 | $ 3,315.80 |

**Figure 1: Tornado diagram of percentage changes to total costs from deterministic one-way sensitivity analysis of parameters with discrepancies for VATU (Zambia)**

**
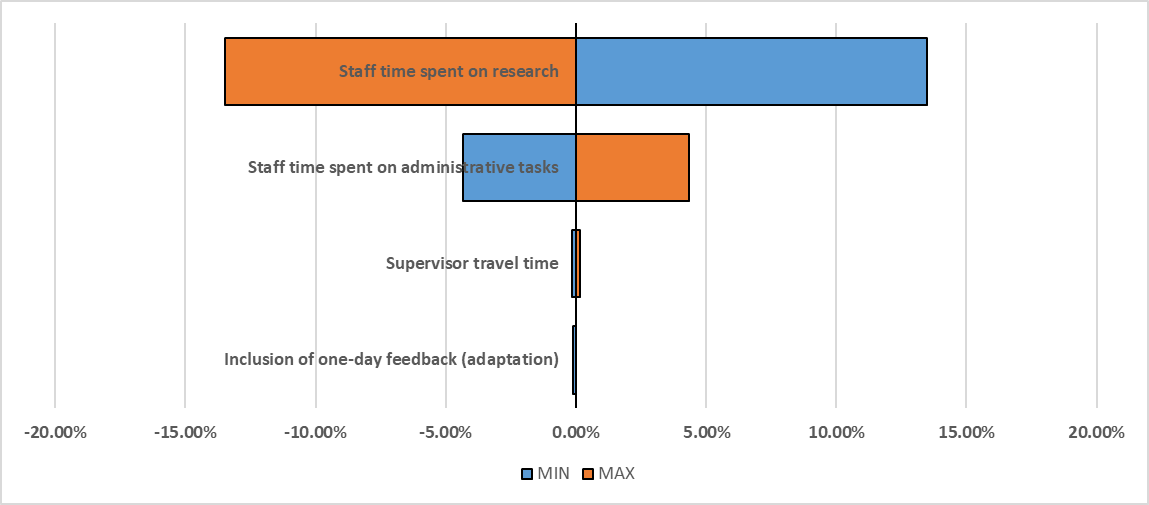
**

**Online Appendix 2: Additional Deterministic Sensitivity Analysis for Indashyikirwa (Rwanda)**

**Table 2: Parameters for Sensitivity Analysis**

|  | **Base Case:**  100% Adaptation /  0% Development | **Scenario 1:**  80% Adaptation /  20% Development | **Scenario 2:**  60% Adaptation /  40% Development | **Scenario 3:**  40% Adaptation /  60% Development | **Scenario 4:**  20% Adaptation /  80% Development | **Scenario 5:**  0% Adaptation /  100% Development |
| --- | --- | --- | --- | --- | --- | --- |
| Start Up Costs: Development (USD) (excluded) | $- | $18,357.66 | $36,715.33 | $55,072.99 | $73,430.65 | $91,788.32 |
| Start Up Costs: Adaptation (USD) | $91,788.32 | $73,430.65 | $55,072.99 | $36,715.33 | $18,357.66 | $- |
| Start Up Costs: Set Up (USD) | $297,659.67 | $297,659.67 | $297,659.67 | $297,659.67 | $297,659.67 | $297,659.67 |
| Implementation Costs (USD) | $2,074,031.31 | $2,074,031.31 | $2,074,031.31 | $2,074,031.31 | $2,074,031.31 | $2,074,031.31 |
| **Total Costs (USD)** | **$2,463,479.30** | **$2,445,121.64** | **$2,426,763.97** | **$2,408,406.31** | **$2,390,048.65** | **$2,371,690.98** |

**Figure 1: Tornado diagram of percentage changes to total costs from deterministic one-way sensitivity analysis due to changes in assumptions of distribution between development and adaptation costs for Indashyikirwa (Rwanda)**

**
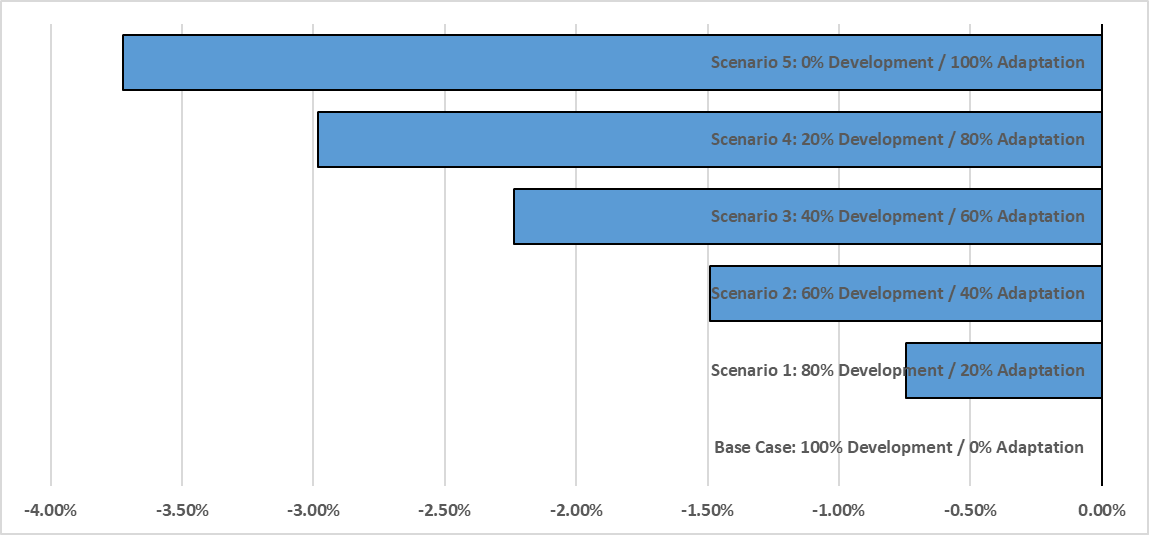
**
